# Exosomal miRNAs as potential biomarkers of cardiovascular risk in children

**DOI:** 10.1186/1479-5876-12-162

**Published:** 2014-06-10

**Authors:** Abdelnaby Khalyfa, David Gozal

**Affiliations:** 1 Section of Pediatric Sleep Medicine, Department of Pediatrics, Comer Children’s Hospital, Pritzker School of Medicine, The University of Chicago, Chicago, IL, USA; 2 Department of Pediatrics, University of Chicago, 900 E, 57th Street, KCBD, 4112, Chicago, IL 60637, USA

## Abstract

Intercellular interactions are essential for basic cellular activities and errors in either receiving or transferring these signals have shown to cause pathological conditions. These signals are not only regulated by membrane surface molecules but also by soluble secreted proteins, thereby allowing for an exquisite coordination of cell functions. Exosomes are released by cells upon fusion of multivesicular bodies (MVB) with the plasma membrane. Their envelope reflects their cellular origin and their surface and internal contents include important signaling components. Exosomes contain a wide variety of proteins, lipids, RNAs, non-transcribed RNAs, miRNAs and small RNAs that are representative to their cellular origin and shuttle from donor cells to recipient cells. The exosome formation cargo content and delivery is of immense biological interest because exosomes are believed to play major roles in various pathological conditions, and therefore provide unique opportunities for biomarker discovery and development of non-invasive diagnostics when examined in biological fluids such as urine and blood plasma. For example, circulating miRNAs in exosomes have been applied as functional biomarkers for diagnosis and outcomes prediction, while synthetic miRNAs in polymer-based nanoparticles are applicable for therapeutics. This review provides insights into the composition and functional properties of exosomes, and focuses on their potential value as diagnostic markers in the context of cardiovascular disease risk estimates in children who suffer from conditions associated with heightened prevalence of adverse cardiovascular disease, namely obesity and sleep-disordered-breathing.

## Cardiovascular disease risk in children: obesity and obstructive sleep apnea as prototypic risk factors

Cardiovascular diseases (CVDs) are the major source of global morbidity and death and more people die annually from CVDs that from any other disease. With the increased worldwide prevalence of obesity, widely regarded as an independent risk factor for a range of chronic diseases including type 2 diabetes and cardiovascular disease [1], a heightened morbidity and mortality from many cardiovascular conditions including hypertension, dyslipidemia, and coronary artery disease has emerged [2,3].

In children, obesity is associated with increased risk for multifaceted derangements in metabolic and cardiovascular function, including endothelial dysfunction (ED) [4-6]. In general terms, overweight children are more likely to prematurely develop ED, hypertension and type 2 diabetes, an array of conditions that would normally be only found in older obese adults [7]. Recently, we have reported that obesity in children is associated with an increased risk for the development of ED, even prior to the onset of hypertension [5]. A recent statement by the American Heart Association has clearly identified the marked increases in long-term cardiovascular risk associated with childhood obesity [8]. Indeed, even short periods of overweight or obesity during childhood have been linked to earlier mortality in adulthood [9]. Therefore, early detection of children at risk for such adverse outcomes would be critically important to enable a more personalized intervention aiming at CVD risk reduction. Although measurement of 24-hour systemic blood pressure, assessment of intima-media thickness and of endothelial function have all been advocated as potentially providing approaches for CVD risk stratification, their implementation in the clinical setting is fraught with objective difficulties that preclude their universal implementation [10,11]. Thus, delineation of more readily detectable biomarkers would definitely be desirable. To this effect, assays of hsCRP have unfortunately failed to deliver with sufficiently predictive precision, thereby reinforcing the need for development of more accurate predictive approaches of CVD risk in pediatric populations [12-14].

## Characteristics of biomarkers

To enable the use of exosomal contents to serve as useful and implementable clinical biomarkers, several conditions need to be met. Indeed, a biomarker must be accurate, sensitive and specific; it needs to be altered in the relevant disease, and be able to discriminate between healthy and diseased subjects; quantification of the biomarker should be reliable and reproducible; the biomarkers should be consistent in different circumstances at different times, and the results from biomarker assays should be easy to interpret. A biomarker for diagnostic purposes should ideally be obtained from readily accessible body fluids, such as blood plasma, urine, and saliva and provide the clinicians with diagnostic information and aid in the medical decision making process [15-17].

Several circulating miRNAs in plasma have been successfully identified as biomarkers for a number of diseases including oncological and non-oncological conditions [18-20], and undoubtedly have come to play increasingly important roles in clinical applications such as disease diagnostics, monitoring therapeutic effects and predicting recurrence in specific patient populations. Specifically, circulating miRNAs have many characteristics of good biomarkers. (1) miRNAs are stable in the circulation and resistant to storage handling, and serum miRNAs are resistant to RNase digestion and other harsh conditions such as extreme pH, boiling, extended storage, and multiple freeze-thaw cycles. (2) Most miRNAs sequences are conserved across species. (3) Changes in miRNA levels in circulation have been associated with different diseases as well as with certain biological or pathological stages. (4) miRNAs levels can easily be determined by various methods [21-24]. Thus far, there are few if any studies on the study of miRNAs in children as biomarkers indicative of potential risk of cardiovascular disease, and such void represents a unique opportunity for the field [25,26].

## Cellular communication

It is now firmly established that communications between cells are essential for appropriate development and function. The classical modes of intercellular communication involve cell junctions, adhesion contacts, and soluble factors that can act upon the same cell where they are produced, or upon neighboring cells, or may even act over long distances in an endocrine manner [27]. Cells can communicate directly with each other through cell-cell contact or at a distance using secreted soluble mediators. In multicellular organisms, cells can communicate via extracellular molecules such as nucleotides, lipids, or proteins. In addition, cells release various types of membrane vesicles into the extracellular space that differ in origin, size, morphology and content to best adapt to their surrounding microenvironment. These extracellular vesicles contain numerous proteins, lipids, and even nucleic acids (Couzin, 2005), and are released in the extracellular space and bind to receptors on other cells, thus modifying intracellular signaling in the recipient cells. Cells may release membrane vesicles into their extracellular environment either by pocketing them off directly from the plasma membrane or through secretion by endocytic compartments [28]. These small, defined vesicles released by cells have been variously termed”exosomes”,”microparticles”,”microvesicles”, and”apoptotic blebs”, and their nomenclature is strictly based on size, density, and membrane composition (for more details see http://www.microvesicles.org/).

Extracellular vesicles contain defined patterns of mRNA, miRNA, long non-coding RNA, and occasionally genomic DNA, and therefore their contributions to cellular trafficking consists in the transfer of genetic information that in turn induces transient or persistent phenotypic changes in the recipient cells [29,30]. Most circulating small RNAs (sRNAs) are contained within lipids or lipoprotein complexes, apoptotic bodies or exosomes that efficiently protect them from degradation by serum ribonucleases. Several reports have now demonstrated that RNAs contained within exosomes can be transferred and modulate the function of the recipient cells [29,31,32].

The important roles of extracellular vesicles in the pathogenesis of various diseases have recently been the object of substantial attention and research. These efforts have been particularly focused on cellular communication in the context of how organized sets of molecules may be involved in many different cellular processes, have the capacity to transmit antigenic information and can be easily recovered from fluids. Another emerging area of discovery that is not related to exosomes, and yet involves mechanisms by which cells relay information to other cells, has been the identification of long, thin, interconnecting membranous bridges that connect neighboring cells through adhesion mechanisms or tunneling nanotubes that can establish direct tubular conduits between the cytoplasms of adjacent cells [33]. Thus, both extracellular and intercellular connectivities are emerging and providing a unique opportunity to explore normal homeostatic processes, disease states, and therapeutics. Here we will review our current understanding on exosomes, and how the latter can serve in the context of cardiovascular disease risk identification and prevention in children.

## Exosome biogenesis and compositions

Exosomes are one of several cell-secreted vesicles, which include microvesicles, ectosomes, membrane particles, exosome-like vesicle’s and apoptotic bodies. Exosomes exhibit pleiotropic biological functions, including immune response, antigen presentation, intracellular communication and the transfer of RNA and proteins. The features and characteristics of the 3 major cell-derived microvesicle types are listed in Table 1.

**Table 1:** Key features of the main extracellular vesicle populations

**Feature**	**Exosomes**	**Microvesicles**	**Apoptotic bodies**
1-Size	40-100 nm	100-1000 nm	1000-5000
2-Markers	CD63, CD9, Alix, and TSG101	Annexin V, integrin, selectin, flotillim-2	Annexin , DNA, histones
3-Cell shapes	Multivesicular bodies fusion with plasmatic membrane	Membrane blebbing	Cell shrinkage and cell death
4-Contents	Proteins, RNA, and miRNA	Proteins, RNA, and miRNA Membrane permeable	Cell organelles, proteins, DNA, RNA, and miRNA
5-Detection methods	FACS with CD68 capture, electron microscopy, Western blot for exosomes enriched markers	FACS and electron microscopy	FACS and electron microscopy
6- Isolation method	Ultracentrifugation (100,000-200,000 g), precipitation, ultracentrifugation with density gradient Immunoprecipitation (ExoQuick)	Ultracentrifugation (10,000- 60,000 g)	No standardized method
7-Mechanism of release	Exocytosis of MVBs	Budding from plasma membrane	Cell shrinkage and plasma membrane blebbing during cell death
References	[34]	[35]	[36]

Exosomes are 30–100-nm diameter membrane vesicles of endocytic origin that are released by most cell types upon fusion of multivesicular bodies (MVB) with the plasma membrane, presumably as a vehicle for cell-free intercellular communication. Exosomes were first described during the 1980s as organelles aimed at removing cell debris and unwanted molecules. For example, during reticulocyte maturation the transferrin receptor and many membrane-associated proteins were shed in small membrane vesicles via an unknown secretory process [37,38], essentially with exosomes functioning as cellular garbage disposals. Since then, exosomes have emerged as important mediators of cellular communication that are involved in both normal physiological processes, such as lactation [38], immune response [39], and development and progression of diseases, such as liver disease [40], neurodegenerative diseases [41], and cardiovascular disease [42]. While the complete array of biological functions played by exosomes remains unclear, they clearly mediate communications between cells and facilitate processes such as antigen presentation as well as trans-signaling to neighboring cells [43].

Exosomes are secreted by many cell types, including B cells [44], T cells [45], dendritic cells [46], mast cells [47], platelets [48], and tumor cells [49]. In addition, exosomes have been found in bodily fluids, including urine and amniotic fluid [50], serum [51], plasma [52], saliva [39], breast milk [39,53], cerebrospinal fluid [54], and nasal secretions [55], suggesting that these microvesicles may be exploited as biomarkers in the diagnosis of disease [56,57].

**Figure 1:**
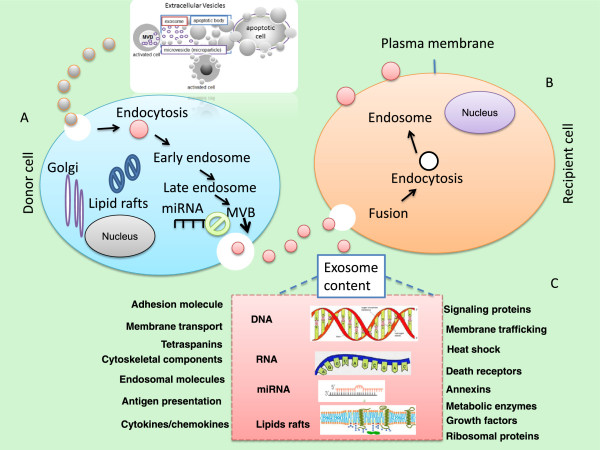
**Schematic representation of extracellular vesicles (EVs) and the transfer of genetic material by exosomes.** These EVs include different types of membrane vesicles (apoptotic bodies [ABs], microparticles/microvesicles, and exosomes), which can be found in body fluids including plasma and urine. The signals of extracellular vesicles can be activated through different steps based on environmental stimuli such as stress or hypoxia. Extracellular signals activate the fusion of multivesicular bodies (MVBs) into the plasma membrane and foster the release of their intraluminal vesicles (ILVs) as exosomes from the donor cells as illustrated in Panel **(A)**. Once the exosomes are released and reach their targets they will start to fuse with the recipient cells **(Panel B)**. Some proteins are directed by the ESCRT (endosomal sorting complex required for transport) machinery to the MVB route. Extracellular vesicles, which are secreted into the extracellular environment, contain functional molecules that can be taken up by recipient cells through mechanisms that include fusion with the plasma membrane, phagocytosis and endocytosis. Panel **(C)** represents the content of exosomes which contains a large array of proteins some of which are involved in membrane transport and fusion (such as RAB proteins and annexins), cytoskeletal proteins, adhesion molecules and tetraspanins, lipid rafts, as well as RNA (mainly miRNA and RNA), and DNA. Exosome membranes are enriched in lipid-based rafts such as cholesterol, ceramide and sphingolipids.

Biochemical and proteomic analysis of exosomes further uncovered that these vesicles contain, besides a common set of membrane and cytosolic molecules, cell-type specific proteins that characterize their functional activity [58]. The mechanisms involved in protein sorting and loading into the exosome are still under investigation, but clearly involve ubiquitin and Endosomal Sorting Complex Required for Transport (ESCRT) machinery in this process. Exosomes are generated as intraluminal vesicles (ILVs) by a mechanism of”reverse budding” of the limiting membrane of late endosomes, which become multivesicular bodies (MVBs) when their lumen contains ILVs. When MVBs fuse with the plasma membrane, their cargo of ILVs is released to the extracellular space, as illustrated in Figure 1. A number of molecules are involved in the biogenesis and secretion of exosomes such as the ESCRT complex which is responsible for the sorting of proteins/cargos in the of MVBs [59]. Recently, a ceramide-dependent mechanism via neutral sphingomyelinase 2 (nSMase2) was proposed to be implicated in the regulation of exosome secretion [60]. Other molecules regulating the docking and fusion of MVBs with the plasma membrane are Rab proteins and SNARE (Soluble NSF Attachment Protein Receptor) proteins [59].

Exosomes can interact with recipient target cells via different mechanisms such as fusion with the plasma membrane or adhesion to corresponding receptors on the plasma membrane [61]. Several lines of evidence suggest that induction of intracellular calcium [61], overexpression of Rab11 or citron kinase [62] as well as a reduction in membrane cholesterol, or inhibition of cholesterol biosynthesis [63], could stimulate the release of exosomes into the microenvironment.

Extracellular membrane vesicles, play important pleiotropic roles in many biological processes, and are enriched in various bioactive molecules, such as growth factors, lipids, membrane receptors, mRNA, miRNA, transcriptional factors. Of note, circulating miRNAs, for example, are released from the parental cells to the extracellular milieu in highly stable forms, and in order to be protected from degradation by extracellular RNases, these cellular miRNAs can be released as soluble ribonucleoprotein complexes or inside cell-derived vesicles. In blood, the majority of cell-free miRNA is associated with the protein Argonaute 2 (AGO2), a main component of the RNA-induced silencing complex (RISC). These AGO2–miRNA complexes protect circulating soluble miRNAs from degradation by RNases present in plasma.

The bioactive molecules contained in exosomes have been suggested to (1) directly stimulate target cells via bioactive lipids or by acting like soluble cell-surface signaling complexes; (2) transfer oncogenic cargo and cancer cell properties to proximate indolent or normal cells; and (3) epigenetically reprogram recipient cells via the transfer of mRNA, miRNAmicroRNA, and transcription factors. Exosomes can be transported between different cells and influence physiological pathways in the recipient cells. Exosome composition varies depending on the cell type of origin. Recent information from different cell type reveals that exosomes may contains a large variety of constitutive elements. Indeed, 4,563 proteins, 194 lipids, 1639 mRNA and 764 miRNA were identified in exosomes [34,64] demonstrating their complexity and potential functional diversity (see below).

### Exosome protein composition

The protein composition of exosomes isolated from different cell sources has now been analyzed by different techniques including proteomics, Western blots and flow cytometry (FACS). These proteins essentially reflect both the cell type from which they are secreted and their endosomal origin, as well as their possible physiological roles and targeting properties [65,66]. Proteomic analysis of exosomes generated from different cell types/lines and biological fluids revealed that exosomes have common characteristic marker proteins on their surface or in their lumen, which are proteins from the cytosol, the plasma membrane, but also from the Golgi apparatus and endoplasmic reticulum [58,66,67] (see Figure 1). As illustrated in Figure 1, the intercellular communication signals can be stimulated based on environmental stimuli. The message (s) transmitted by these intercellular communications may include growth, division, survival, differentiation, stress responses, and apoptosis signaling. The apoptotic bodies can be released as blebs of apoptotic cells, and they are characterized by phosphatidylserine externalization and contain fragmented DNA. Interestingly, exosomes can carry complex biological information consisting of mRNAs, miRNAs, as well as soluble and transmembrane proteins between cells.

One important challenge in the study of exosomes has been the lack of robust, reproducible isolation methods to obtain highly pure and well-characterized vesicular populations. Several strategies have been used for exosome isolation based on their size. There are 4 different methods that have been proposed to isolate and characterize exosomes including: (1) ultra-centrifugation [68], (2) ultra-filtration [69], (3) immunoprecipitation technologies with the use of antibody-loaded magnetic cell beads [70], and more recently (4) ExoQuick precipitation methods (System Biosciences, Mountain View, CA, http://www.systembio.com, and Life Technologies, Foster City, CA, http://www.lifetechnologies.com), based on size-based precipitation approaches, a process that is relatively short, requiring less than 2hours, and proven to be efficient, reliable and reproducible when compared to the other methods.

Exosomes can be produced by the endocytic and exocytic activity in physiological and physiopathologic conditions [71,72]. A number of proteins families were identified in exosomes and many of these, such as heat shock proteins, annexins, and proteins of the Rab family, are primarily involved in intracellular assembly and trafficking of exosomes, and may not be required further after the vesicles are secreted [71]. Other ubiquitous proteins are involved in antigen presentation, as they can bind antigenic peptides and participate in loading peptides onto MHC molecules [73]. Tetraspanins are one of the most abundant protein families that is found in exosomes, consisting of a large number of transmembrane proteins. Several members of this family, such as CD9, CD63, CD81 and CD82 are highly enriched in exosomes. Tetraspanins interact with many protein partners including MHC molecules and integrins, suggesting that they are involved in the organization of large molecular complexes and membrane subdomains [66,74]. Integrins are another group of abundant proteins detected in exosomes, where they exist as heterodimers of α and β subunits, and function as adhesion molecules facilitating binding to the extracellular matrix [75].

### Exosome lipids composition

The lipid composition of mast cell-derived exosomes includes lysophosphatidylcholine, sphingomyelin, phosphatidylcholine, phosphatidylserine, phosphatidylethanolamine cholesterol and diglyceride [76]. It has been indicated that phosphatidylserine is a key determinant for the interaction of membrane vesicles with target cells although oxidized lipids which may also indicates that play a role. Phosphatidylserine and microparticles of different cellular origin can bind to platelet CD36 scavenger receptor, leading to increase ADP-dependent platelet activation and augmented thrombosis in mice [77]. The phosphatidylserine-moiety of endothelial microparticles can interact with endothelial phosphatidylserine receptor in an annexin I–dependent manner to prevent endothelial apoptosis [78]. It seems that when comparing these lipids with their parent cells, membranes of extracellular vesicles are enriched with phosphatidylserine, disaturated phosphatidylethanolamine, disaturated phosphatidylcholine, sphingomyelin, ganglioside GM3, and cholesterol [79,80]. These enriched lipids as well as lipid-raft-associated proteins in vesicular membranes provide extracellular vesicles with stability and structural rigidity [81,82].

### Exosome miRNAs composition

MicroRNAs (miRNAs) are non-coding RNA (19–22 nucleotides) that post-transcriptionally regulate gene expression by base-pairing with the 3' untranslated region of complementary messenger RNA targets. As mentioned above, miRNAs were found to be present in human plasma and serum in a remarkably stable and cell independent form, and their discovery has led to renewed interest in their use as novel non-invasive biomarkers in the context of physiological and pathophysiological conditions, including cancer and cardiovascular disease [83-87]. In addition to the classical mechanisms such as direct cell-cell contact or chemical receptor-mediated events, recent studies demonstrated that the transfer of exosome-derived unique miRNAs to recipient cells is an alternative mechanism enabling gene-based communication between cells [60]. Thus, RNA containing exosomes may represent alternate pathways of cellular communication with significant implications in the modification of cell phenotypes in response to homeostatic pertubations. As such, specific miRNAs such as let-7, miR-1, miR-15, miR-16, miR-151 and miR 375 which play role in angiogenesis, hematopoiesis, exocytosis and tumorigenesis have been reported in exosomes [74].

## Exosome functions

To understand the biological function of exosomes, a number of transcriptomic and proteomic profiling analyses have been performed [71,67,79]. Exosomes can activate or inactivate different pathways on surrounding or remotely-located cells, depending on their molecular composition, which is influenced by the activation state of the secreting cell [88]. The assessment of biological functions of exosomes showed that they can deliver specifically their cargo from the donor to recipient cells. The molecular composition of exosomes reflects the specialized function (s) of their original cells. Through their ability to bind target cells, they are likely to modulate selected cellular activities, such as vascular homeostasis, and antigen presentation. The presence of exosomes in blood and tissues *in vivo*, suggests their participation in physiological and/or pathological processes, such as stimulation of regulatory T-cell lymphocytes, induction of protection against allergic sensitization, induction of cytokine production, and induction of tumor growth [39,66,89-92].

## Exosomes and endothelium

Exosomes are released from endothelial cells [93] and cardiomyocytes [94,95]. Gupta and colleagues showed that the levels of exosome-mediated secretion of HSP60 from adult cardiomyocytes were increased three-fold in mild hypoxia [95], and Waldenstrom and colleagues explored the mRNA content of exosomes released from cardiomyocytes under normal conditions, and identified 1,520 mRNA by microarray analysis [94]. In addition, proteomics analysis of endothelial exosomes revealed about 1,354 distinctive proteins, while microarray analysis revealed 1,992 mRNA transcripts, and there were 21 of these transcripts in endothelial exosomes that were altered in their abundance due to hypoxia [93].

Studies of miRNAs are not only providing sources of disease biomarkers [20], but also enabling greater understanding of cardiovascular development under normal conditions and of remodeling processes in disease. In particular, miR-1, miR-133, miR-208, and miR-499 have been reported to be highly expressed in cardiac tissue from the early stages of heart development, regulating cell proliferation, differentiation, and apoptosis, as well as playing critical roles in several cardiac diseases [96]. Recent studies of circulating miRNA-133a levels showed levels were increased upon the onset of acute myocardial infarction, and also elevated with unstable angina pectoris and Takotsubo cardiomyopathy, even in the absence of elevation of serum creatine phosphokinase or cardiac troponin, two standard markers of acute cardiac insults [97].

Several reports using primary rodent cardiomyocytes (*in vitro*) have provided evidence of exosome secretion [95,98]. For example, Cheng and colleagues [99] detected significantly higher levels of miR-1 and miR-208 in urinary exosomes derived from acute myocardial infarction **(**MI) patients and in the circulating blood of rats after acute MI. Furthermore, the cardioprotective miR-214 that is secreted via exosomes from human endothelial cells has been shown to be up-regulated in the heart after acute ischemia and also altered in the plasma of coronary artery disease patients, indicating the severity of the disease. Recent work from several laboratories including ours has identified alterations in specific microvesicles in the plasma that not only exhibit severity-dependent changes but also provide suggestions as to the cellular sources of such microvesicles and their potential implications in cardiovascular disease in children [100-104].

## Exosome transfer miRNAs

As mentioned above, exosomes have been found to contain different RNAs, including mRNAs, miRNAs, and long non-coding RNA (lncRNAs). In mammals, miRNAs can be transported by high-density lipoproteins (HDLs) and be delivered to recipient cells to modulate their function [105]. Inhibition of neuronal sphingomyelinase 2 (nSMase2), [32,106], increase the amount of miRNAs exported to HDLs by blocking the release of miRNA-loaded exosomes. Thus, nSMase2 and the ceramide pathway might regulate different but coordinated pathways of miRNA secretion. Argonaute 2 (AGO2) is considered the key effector protein of miRNA-mediated silencing and can form circulating ribonucleoprotein complexes with miRNAs [107,108].

The transfer of intact mRNAs from exosomes to target cells may alter the phenotypic features of recipient cells. Exosome-mediated RNA transfer is believed to be an effective method for cell signaling and the exosomal RNA will certainly impact biological processes in the recipient cells [32,60,109]. Exosomal RNAs have been implicated in many exosome-mediated biological functions [110]. In addition, exosomes are natural carriers of exogenous siRNA to human cells in vitro [111]. Therefore, the ability of exosomes to naturally carry and deliver RNA between cells could be a useful method to deliver therapeutic short interfering RNA (siRNA) to the desired target cells. Studies have shown that the packaging of RNAs into exosomes is selective because the RNA profiles in exosomes do not fully reflect the RNA profiles observed in the parental cells [29,31]. While exosomes have been shown to play functional roles in recipient cells, the RNA content of the exosomes may provide unique molecular signatures for disease diagnosis and prognosis [112]. It has been reported that exosomes from diseased individuals contained RNAs that were not found in healthy subjects [50,112]. Recently, the role of miRNAs in inflammation and metabolic and cardiovascular diseases has been highlighted [113-115].

## Exosome and the endothelial barrier

Endothelial dysfunction (ED), an early risk marker of cardiovascular disease, refers to a loss of normal homeostatic function in the blood vessels, and is characterized by altered vasodilatory and vasoconstrictive functions and inflammatory activity [116]. ED is an early and sustained process involved in the development of vascular complications related to dyslipidemia, and cardiovascular disease such as hypertension, coronary artery disease and chronic heart failure [117,118]. In healthy subjects, the vascular endothelium, which forms the barrier between blood and the surrounding tissues, is known to efficiently respond to stress signals like hypoxia and inflammation by adaptation of cellular physiology and the secretion of cytokines and growth factors that recruit endothelial progenitor cells or cells that play a role in the innate immune response [119]. Also, it has been reported that endothelial cells secrete exosomes [120,121] and several reports show that endothelial cells can also be targeted by exosomes derived from different cell types [122].

The tight junction complex is critically involved in the exchange of ions, solutes, and cells that travel across paracellular spaces. Tight junctions regulate the formation of apico-basal polarity and control proliferation, gene expression, or cell differentiation through signaling pathways that are activated by tight junction components [123]. Several proteins have been identified to be associated with tight junctions [124] such as occludin (OCLN) and claudins (CLD), and these are transmembrane spanning proteins forming the intercellular adhesions via hemophilic and heterophilic interactions. Zonula occludens-1 (ZO-1), ZO-2, ZO-3, or cingulin, are located mintracellularly and anchortransmembrane proteins with the actin cytoskeleton [123]. Tight junction components, such as occludin, claudins, or junctional adhesion molecules, are linked to the actin cytoskeleton by ZO proteins, thereby enabling these proteins to constitute major stabilizing factors of tight junctions. Accordingly, ZO proteins have been shown to be essential for tight junction formation [125]. Recent studies now show that exosomes from patients with cardiovascular disease or from endothelial cells exposed to stressful conditions can disrupt endothelial tight junctions and reflect the extent of endothelial dysfunction or of repair potential [126-134].

**Figure 2:**
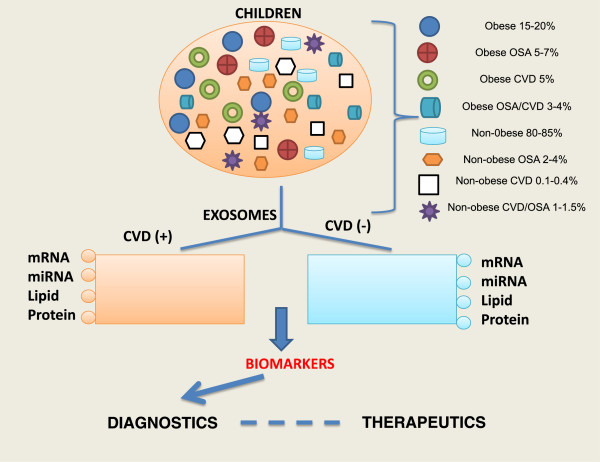
**Schematic diagram illustrating the potential use of exosomal miRNAs to identify children at risk for cardiovascular disease (CVD (+)).** Children with obesity, OSA, or both may be at increased risk of CVD but currently their identification is difficult. Exosomes isolated from children with CVD (+) will differ from those children that do not exhibit increased CVD risk (CVD (−)). Identification and validation of candidate exosomal miRNAs can then be used as biomarkers for diagnostic purposes and potentially provide insights into therapeutic strategies.

In the context of OSA, in depth exploration of the exosomal content is lacking. Initial work on microvesicles and OSA would definitely justify further efforts since a substantial degree of correlations between microvesicles from different cellular sources and cardiovascular outcomes including ED have been identified [135-145]. Similar observations are applicable in the context of obesity, whereby specific microvesicles appear to be closely associated with metabolic or cardiovascular outcomes [135-146]. Furthermore, children with obesity, OSA, or both may be at increased risk of CVD, but their identification is currently difficult. We postulate that exosomes isolated from children with CVD (+) will differ from those children that do not exhibit increased CVD risk (CVD (−)). Identification and validation of candidate exosomal miRNAs can then be used as biomarkers for diagnostic purposes, and potentially provide insights into therapeutic strategies. We propose as illustrated in Figure 2, a schematic diagram illustrating the potential use of exosomal miRNAs to identify children at risk for cardiovascular disease.

## Exosomes in diagnosis and therapy

As discussed above, circulating cell-free nucleic acids have promising potential as non-invasive diagnostic markers for pathological processes such as chronic inflammation, cancer and cardiovascular disease [147,148]. More recently, have emerged as possible vehicles for the delivery of therapeutic molecules, and have mainly been investigated in the context of cancer therapy on account of their T-cell stimulatory capacity. For instance, exosomes loaded with tumor antigens have been shown to stimulate CD4+ and CD8 + T cell lmphocytes, and exosomes from in vitro cultured antigen presenting cells (APCs) administrated in *vivo* can induce T-cell lymphocyte responses resulting in inhibition of tumor growth [149,150]. The first two promising phase I trials in human cancer have been published [151,152] using exosomes from monocyte-derived DCs loaded with tumor antigens. The use of exosomes in cardiovascular diseases is being now intensively explored but unlikely to be readily applicable in children.

Despite the relative facility to collect exosomes from biological fluids, the actual use of exosome-derived proteins or miRNAs as biomarkers is, however, not yet implemented in clinical practice. Minimally invasive diagnostics (based on analysis of blood) or non-invasive diagnostics (using urine and saliva samples) are superior alternatives to traditional needle or excision biopsies due to the reduced patient pain and inconvenience, and greater speed and lower cost of analysis. Indeed, several companies have initiated development of the exosome-based diagnostics within the last three years. For example, a sensitive diagnostic of prostate cancer based on analysis of exosomal proteins was recently launched (http://www.CarisLifeSciences.com). Exosome diagnostics is developing biofluid-based molecular diagnostic tests for use in personalized medicine. The main focus thus far is on oncology or neurodiagnostics through exosome-based technology platforms (http://www.ExosomeDx.com; http://www.HansaBioMed.eu). We propose that similar to some of the proteomic biomarkers emerging in the context of pediatric sleep apnea and cardiovascular disease [153-157].

## Conclusion

Cells communicate with each other via extracellular molecules, such as nucleotides, lipids, miRNA, or proteins. These molecules can then be released extracellularly by cells in microvesicles and bind to receptors on other cells, thus inducing intracellular signaling and modification of the intracellular physiological state of recipient cells. Exosomes have been isolated and characterized from many different biological fluids such as blood components, urine, amniotic fluids, malignant effusions, breast milk and bronchoalveolar lavage fluid and contain an array of proteins and lipids as well as genetic material such as mRNA and miRNA. The unique opportunities for the discovery and use of exosomes contents as clinical biomarkers of cardiovascular disease risk in obese children or in other groups of children with diseases associated with cardiovascular morbidities such as pediatric sleep apnea appears to be warranted. Indeed, the study of exosomes in the context of pediatric obesity or OSA will illustrate novel mechanisms of cell–cell and organ–organ communication, identify novel biomarkers of the disease and its complications, and potentially aid in the development of novel therapeutics.

## Competing interests

The authors declare that they have no competing interests.

## Authors’ contributions

AK provided the conceptual design of the project, writing and editing final version of the manuscript. DG participated in writing and editing final version of the manuscript. Both authors read and approved the final manuscript.
